# A look at the free-viewing paradigm in eye-tracking research to assess positive attentional bias

**DOI:** 10.3389/fpsyt.2025.1659072

**Published:** 2025-12-10

**Authors:** Thomas Suslow, Dennis Hoepfel, Taavi Wenk, Anette Kersting, Vivien Günther

**Affiliations:** Department of Psychosomatic Medicine and Psychotherapy, University of Leipzig Medical Center, Leipzig, Germany

**Keywords:** attention, emotion regulation, eye-tracking, free viewing, positive attentional bias, positive mood, time-course of bias

## Abstract

Attention orientation toward positive stimuli may have mood-protective or mood-enhancing effects. Eye-tracking is an increasingly administered method to assess biased attention allocation and maintenance. In the present paper, we point to an underestimated but reliable method of eye-tracking research for measuring positive attentional bias and its temporal dynamics: the free-viewing paradigm. To date, few free-viewing eye-tracking studies have specifically examined positive attentional biases in healthy individuals. Against this background, we summarize findings from clinical and subclinical eye-tracking research using free viewing in healthy control groups. We discuss the observed time courses of positive attentional biases during experimental trials, which vary depending on type and number of presented stimuli, and make recommendations on which experimental conditions appear to be favorable for capturing dynamic time courses of positive attentional biases. We identify various individual difference factors that may influence the magnitude of positive attentional biases and should be considered in future studies. Time course analyses of eye-tracking data offer the opportunity to learn more about the time of onset and extent of increase in attention to positive information during free viewing and their relationships to individual difference variables. Directions for future research on positive attentional biases are discussed.

## Introduction

From an evolutionary perspective, positive thinking can be seen as a survival mechanism, which helps people to navigate through difficult situations and increase the likelihood of achieving favorable outcomes (1). Healthy individuals experience positive emotions more frequently than negative ones in everyday life (2). According to cross-national data, humans show a general preponderance of positive emotion over negative emotion, with the possible exception of very poor societies (3). Higher positivity ratios are associated with better mental health (4). Various cognitive positivity biases may contribute to this predominance of positive emotions. A positive bias can refer to the tendency to recall positive aspects of past events more vividly than negative ones (5), the tendency to be optimistic about one’s future (6, 7), or self-serving attributional tendencies, making more internal, stable, and global attributions for positive than for negative events (8). Another form of positivity bias is observed in the area of ​​attention processes: individuals appear to prioritize and pay more attention to positive or rewarding stimuli compared to neutral ones (9). A positive attentional bias may have beneficial effects on mood, stress resilience, and social contact (10–12).

An important feature of attention is its selectivity, i.e., cognitive resources are focused on certain aspects of the environment rather than on others (13). Selective processes can be observed at early and late stages of perception. In the area of emotion perception, automatic vigilance for negative and especially threatening stimuli has been documented during early information processing (14, 15). Clinical research on attentional biases has shown that individuals with anxiety disorders exhibit a particularly facilitated detection and orienting to threat-related stimuli compared to healthy individuals (16). In the last decades, clinical studies of attentional biases have frequently relied on reaction time tasks such as the dot-probe task (17), the emotional Stroop task (18), or the face-in-the-crowd task (19). These tasks are indirect measures of attention, which require a motor response and depend on speed-accuracy criteria. They rely on reactions to stimuli measured at a specific point in time. A serious methodological problem of reaction-time measures lies in the poor reliability of the attention parameters derived (20–22).

Recently, eye-tracking technology has gained increasing popularity in various research areas (23, 24). Eye-tracking allows continuous recording of eye movements and represents a direct method to assess attention allocation, as direction of eye gaze and focus of attention are often closely linked (25). The highest visual quality is achieved within a small foveal region at the center of gaze that covers about 2° of visual angle (26). Where the eyes look determines which visual information is focused on and perceived in more detail. Eye-tracking has become one of the most important experimental methods in the study of attentional biases in mental disorders (27–31). It can be combined with various experimental paradigms such as the dot probe (32) or the face-in-the-crowd task (33), e.g., to assess early and late attention allocation or scan paths. It is interesting to note that when eye movements are registered and analyzed during dot probe tasks, eye-tracking and traditional reaction-time based attention parameters are largely unrelated (34–36). It appears that these different attention bias measures could reflect different stages of processing or attention types (overt vs. covert) (37, 38). Therefore, registration of eye-movements during the dot probe task does not appear to be a kind of substitute for reaction times in determining attention allocation. However, eye-tracking indices of late attentional processes such as bias scores based on dwell time or fixation frequency tend to yield higher reliability coefficients (internal consistency) than traditional reaction-time based bias scores (34, 36). In the dot probe task, eye-tracking indices of early attentional processes, such as the proportion of first fixations or the first fixation latency bias, also show poor reliability (34).

A frequently used paradigm in eye-tracking research to measure attentional biases in emotion perception is free-viewing. In the next section, we will introduce the free-viewing paradigm and discuss methodological features such as task instructions, type and number of stimuli used and the reliability problem of the attention parameters derived from the task. In our paper, we then present the evidence for a positive attentional bias in healthy individuals as reported in clinical studies, since there is little specific research on positive attentional bias in healthy individuals. The literature discussed comes primarily from recent meta-analyses on attention bias in depression and anxiety (28–30, 39) and additional searches in the databases Web of Science and PsychINFO. Afterwards, we point out various individual difference factors that can influence the magnitude of positive attentional biases during free viewing. Finally, we deal with the time course of visual attention during free viewing, since little is known about the onset of positive attentional biases but fast gaze orientation toward positive stimuli could be important for efficient mood stabilization and repair.

## The free-viewing paradigm

The free-viewing paradigm, sometimes also called passive viewing paradigm, refers to a research approach where participants are asked to observe one or more stimuli (such as images, words, or videos) without any specific task. This setup enables the investigation of spontaneous gaze behavior and attention allocation. Free-viewing tasks are relatively simple to implement, making them suitable for a wide range of research questions and study populations including, for example, infants, apes, and dogs (40–42).

In a laboratory setting, participants’ only task is to observe the stimuli shown on the monitor. This research methodology contrasts with traditional experimental paradigms where participants are instructed to process visual stimuli in a specific way (e.g., judge the age of individuals shown) while viewing the stimuli. Free-viewing tasks differ from traditional reaction time tasks assessing attention processes, where, for example, it is necessary to respond as quickly as possible to the location of a dot that replaces one of two stimuli (as in dot probe tasks (17)), to determine the color of a word as quickly as possible, while ignoring the word’s meaning (as in emotional Stroop tasks (18)) or to look at a series of faces and search for a discrepant emotional expression as quickly as possible (as in face-in-the-crowd tasks (19)).

The exact instructions in free-viewing tasks can vary across studies. For example, in some investigations, participants were asked to look at the images in any fashion they wished (43), to view the images naturally, with no further requirements (44), or to look attentively at the stimuli presented (45). Future research has to clarify whether instructions that encourage more attentive viewing could elicit more exploratory, information-seeking gaze behavior compared to instructions that emphasize freedom and spontaneity of viewing. Attentive viewing instructions may increase exploratory behavior, depth of stimulus encoding, and accelerate eye movements compared to more passive viewing instructions (46).

In free-viewing tasks, either a single stimulus (e.g., face or image) can be presented or multiple stimuli (e.g., 2, 4, or 16) can be displayed simultaneously. When only one stimulus is shown, it can be examined which details of the stimulus are viewed first and for how long (47, 48). The presentation of multiple stimuli offers the opportunity to investigate processes of early and late preference for one stimulus category over another. Early attention allocation can be assessed through parameters such as location (or probability) of first fixation or first fixation latency, i.e., the time it takes to fixate on a stimulus. Parameters like dwell time or fixation duration measure processes of sustained attention allocation and refer to the length of time the gaze remains focused on a specific stimulus. A serious methodological problem of early attention parameters derived from multiple-stimulus free-viewing tasks lies in their poor reliability, i.e., low internal consistency, split-half and test-retest reliability (34, 49, 50). An important factor contributing to these poor reliability results for early parameters could be culturally shaped initial gaze behavior, which is directed to the left in Western and to the right in Eastern cultures, when one should read or look at something. In contrast, dwell time and fixation duration measures derived from multiple stimulus free-viewing tasks yield in general adequate to good internal consistencies and split-half reliabilities (34, 49–51) and, in many cases, acceptable test-retest reliabilities (49, 50, 52). Reliable, psychometrically sound attention parameters are necessary for promoting our understanding of the attention processes implicated in psychopathology (53).

Researchers wanting to use free-viewing tasks should ensure that study-relevant contents or stimulus categories are not primed by prior presentation of another experiment or prior completion of questionnaires. Primed features can automatically guide attention to stimuli with the same features as the prime in subsequent displays, no matter whether prime features were implicitly or explicitly encoded (54).

## Positive attentional bias as assessed during free viewing: the effect of depression, mood states, individual differences, and age

Free viewing has been widely used in clinical research to investigate decrease in positive attentional bias in patients with depressive disorders (30, 39). In addition to an increased attention for dysphoric information, clinically depressed patients are characterized by reductions in attention maintenance for positive stimuli compared to healthy individuals. The two forms of attentional distortions in depression, i.e., more sustained attention to dysphoric stimuli and reduced attention allocation to positive stimuli, can occur independently of each other. These attentional biases have been demonstrated separately in experiments in which a sad (or happy) face was presented alongside a neutral face (44, 55). The studies in the field focused on group differences between depressed and non-depressed individuals and hardly addressed questions concerning the magnitude of attentional preferences for positive over negative information in the non-depressed control groups. An inspection of the data from the relevant free-viewing studies provides evidence that the gaze of healthy individuals (at least descriptively) remains longer on positive than on negative stimuli - regardless of whether faces (shown for 3 to 10 seconds) or images (shown for 30 seconds) were presented in the experiments (faces (44, 51, 56–59), images (60, 61) – but see (55, 62) for discrepant results). The two studies with discrepant results were both face-based and presented only two faces. Moreover, the proportion of women in these investigations was slightly lower (53% and 56%) than in the other studies (61-75%) (44, 56–59). Thus, the detection of a positive attentional bias may be more difficult when pairs of faces are presented than when four or more faces are used (56–58). A low proportion of women in a sample could also make it more difficult to detect positive bias effects. Decreases in sustained attention for positive information during free viewing have also been observed in dysphoric or mildly depressed compared to non-depressed individuals (63, 64).

In research on biases in sustained attention allocation or attentional preferences, indices were used that are based on dwell time, fixation duration or number of fixations. In contrast to fixation duration, dwell time includes also the time spent on a specific area of interest during gaze movements. In many studies, the analysis for determining attentional biases was based on the mean dwell or fixation time (in seconds or milliseconds) to emotional and neutral stimuli, which were then entered into statistical analyses (57, 58, 61, 63–65). In several other studies, relative bias scores were calculated, for example, by subtracting the fixation time to neutral faces from that to a specific emotion face category (44, 55), dividing the fixation time to a specific emotion face by the fixation time spent on a multiple-stimulus array (59), calculating the percentage of time on a specific emotion category compared to all faces (60) or dividing the fixation time to a specific emotion face by the fixation time spent on the specific emotion face and the paired neutral face (34). Although raw dwell time or fixation duration values ​​are often used in eye-tracking based attention bias research, it seems advisable to compute relative bias scores in the case of studies that examine attention to different emotions and present only two stimuli per trial (an emotional combined with a neutral stimulus). Bias scores that consider the total fixation time for both stimuli may improve here the comparability of the magnitude of attention allocation between emotion categories.

Mood states can influence attention allocation in non-depressed individuals during free viewing. Increases in happy mood after positive mood induction was found to be linked to enhanced attentional deployment to happy faces (66). Increased negative mood (in response to negative mood induction) is associated with increased attentional deployment to positive images (43, 67). The latter finding suggests that heightened attention to positive stimuli could be part of an emotion regulation strategy related to mood repair.

Various other individual difference factors can influence the magnitude of positive attentional biases during free viewing. Trait happiness and life satisfaction were found to be positively associated with attention maintenance on positive compared to neutral scenes, irrespective of category, i.e. achievement, social, and primary reward (68). These effects were especially prominent during the later phases of sustained viewing. The personality trait of alexithymia, which is characterized by difficulties identifying and expressing one’s emotions, could attenuate positive attentional biases. Surber et al. (69) observed in non-alexithymic individuals an attentional preference for happy over angry and neutral facial expressions, whereas in alexithymic individuals no attentional preferences were determined. Experiences of maltreatment during childhood may affect the perception of positive content in adulthood. Attentional preference for positive over other emotional faces during free viewing was found to be diminished in women with experiences of physical and emotional abuse in childhood (70). Reductions in attentional preference for positive stimuli may be one pathway through which experiences of severe abuse increase the risk for the development of depressive symptoms and affective disorders.

There are indications that age might be associated with heightened orientation toward positive stimuli during free viewing (71). According to Faul et al. (72) the emergence of the positivity effect in older adults could be related to absence of depressive symptoms and reappraisal regulatory preferences. Thus, in healthy older people preference for using reappraisal in daily life could be related to an increased positive attentional bias.

## Time course of visual attention to positive information

There are eye-tracking studies based on free-viewing tasks that have examined changes in attention allocation not only as a function of emotional quality of stimuli, but also of time. Some of these studies provide information on the time course of attention to positive stimuli compared to other simultaneously shown emotional or neutral stimuli in healthy individuals. Kellough et al. (60) presented happy, sad, threatening and neutral images for 30 seconds to depressed and never depressed individuals. The never depressed individuals were young university students (with an age range between 18 and 21 years; 47% were women). Stable differences between study groups in fixation time were observed for dysphoric and positive faces over time. In the total sample, there was a strong initial attention to threat images during the first 5 seconds of presentation that decreased substantially during the next 5 seconds. In contrast, attention to positive images was initially less pronounced but tended to increase during the experiment. Arndt et al. (64) also used positive, dysphoric, threatening, and neutral images showing them simultaneously for 10 seconds to dysphoric and non-dysphoric individuals. The non-dysphoric individuals were university students (with a mean age of 21 years; only women were included). In the non-dysphoric group, during the first two seconds the longest fixation times were observed for threat images, which decreased subsequently. In contrast, the positive images were viewed less during the first four seconds compared with the threat images, but thereafter there was a steep increase in fixation time for positive images. After eight to ten seconds, positive images were looked at much longer than the other image categories.

In the study of Waechter et al. (34), two faces from the same model were presented simultaneously (a happy, angry, or disgusted expression combined with a neutral one) for 5 seconds to individuals with high and low social anxiety. Social anxiety had no effect on attention but an emotion by time interaction was observed. Study participants were university students (with a mean age of 19 years; 67% were women). Proportion of viewing time was analyzed in 500ms intervals. When viewing anger-neutral face pairs, participants showed a significant attention bias toward anger faces from 501 to 2.500ms. When looking at disgust-neutral pairs, a bias toward disgust faces was found only between 501 and 1.500ms. In the last four 500ms intervals participants looked longer at the neutral compared to the angry or disgusted face (at a descriptive level). When viewing happy-neutral pairs, an attentional bias toward happy faces was observed from the second interval (i.e., 501-1.000ms) to the last interval (i.e., 4.501-5.000ms). In the study of Byrow et al. (65), socially anxious and non-anxious individuals were presented two faces of the same model (a happy or angry expression combined with a neutral one) for 1.5 seconds. The non-anxious individuals were university students (with a mean age of 26 years; 48% were women). Healthy controls showed more fixations on emotional than on neutral faces. No differences in percentage of fixations were observed between happy and angry faces. Fernandes et al. (73) compared visual attention of high- and low-socially anxious individuals showing two faces of the same model (combinations of happy, angry, and neutral expressions) for 1.5 seconds. The low anxious individuals were university students (with a mean age of 22 years; 63% were women). In the low anxiety group, there were no differences in dwell time between angry and happy faces, but dwell time decreased for both emotional qualities over the 1.5 seconds. Gamble and Rapee (74) presented two faces from the same model (a happy or angry expression paired with a neutral one) for 5 seconds to social phobic and non-phobic individuals. 43% of the non-phobic individuals were university students and 57% were community volunteers (with a mean age of 36 years; 43% were women). In the healthy control group, attention for happy faces (compared to neutral faces) was higher compared to attention for angry faces throughout the five 1-second intervals of the experiment. Soltani et al. (75) presented sets consisting of happy, sad, threatening, and neutral faces of different models for 8 seconds to depressed and never depressed individuals. The never depressed individuals were university students or community members (with a mean age of 24 years; only women were included). Already in the first 2-second interval, the never depressed subjects looked at the happy faces the longest. In the never depressed individuals, attention to happy faces increased continuously over the four intervals.

To summarize, the results of the studies based on (four) images indicate an initial attention allocation to threat stimuli (60, 64). Both studies are based on samples of young university students. In the study with a long trial duration, i.e., 30s (60), a longer initial attentional prioritization of threatening images was found compared to the study with a short trial duration, i.e., 10s (64). Possibly, the total period of time available to the participants for observation could play a role in the temporal development of attention allocation. However, across both studies (60, 64) there is evidence that positive images are initially less attended but subsequently they receive more attention. The findings of the face-based investigations show a different picture of attention allocation over time. When four faces are simultaneously presented positive faces are viewed the longest already in the first two seconds followed by a further increase in attention (75). As in the image-based investigation of Arndt et al. (64) study participants of Soltani et al. (75) were young, female and at least in part university students so that differences in the findings on the time course of attention should not be due to the variables age or biological sex. It is interesting to note that when one angry or disgusted face is presented together with a neutral one the initial attention bias toward the threatening (or hostile) face is no greater than toward a happy face (34). Subsequently, anger and disgust faces can lose attention, whereas happy faces may continue to receive more attention than neutral faces over time. There is even evidence that happy faces (in pairs with neutral faces) receive more attention than angry faces in the first second of presentation (74). This early prioritization of positive faces was observed in a sample of individuals who were on average in their mid-thirties and thus somewhat older than the participants in the other studies (34, 60, 64, 65, 73, 75). This raises the question of whether, with increasing age during young adulthood, the attentional prioritization of positive faces might start earlier in the perception process. None of the above cited free-viewing eye-tracking studies on attentional biases administered dynamic stimuli (dynamic facial expressions or video clips).

## Discussion: conclusions and directions for future research

Considering the above findings, the free-viewing paradigm appears generally well suited to capture positive attentional biases in healthy individuals. However, the number, type and duration of the presented stimuli appear to have a substantial influence on the occurrence of the bias. Our analysis of the literature shows that, to date, little research has been conducted that focused on the analysis of the temporal development of positive attentional biases in non-patients. The existing studies were based on samples consisting entirely or partly of university students. This limits the generalizability of the results on the time-course of positive attentional biases and indicates a need for future research to also examine less educated and older individuals. Attentional preferences for positive environmental stimuli could be important determinants of positive affect (10, 12), which should be better understood in terms of their conditions of occurrence. Attention orientation toward positive stimuli could serve as a relatively automatic, low-effort regulatory strategy that promotes mood (68). Directing and holding one’s attention on positive facial expression could act as one mechanism, by which positive affect is elicited or enhanced in everyday life. Processes of emotional contagion might play a crucial role in inducing positive affective reactions during the perception of happy facial expression. Emotional contagion describes the phenomenon that people automatically mimic and synchronize their emotions with those of another person (76) and is assumed to occur through facial mimicry and feedback (77). Receivers imitate senders’ emotional displays in emotional mimicry, even if these are only of short duration (78). Facial feedback from such mimicry processes can activate the corresponding emotional state in receivers. The perception of happy facial expressions can elicit positive feelings in the receivers through facial muscle activity (79).

Importantly, internal consistency and split-half reliability of positive attention bias scores (dwell times) for single time intervals were found to be adequate to good for images as well as faces - except for the first two seconds (50). Individual differences in the propensity to look at positive stimuli are rather stable across contents and across a two-months period (80). In previous eye-tracking studies, reliability data on attention bias parameters were rarely provided, but this should always be done in future studies in order to be able to assess the solidity of the reported findings.

When selecting the stimuli for free-viewing tasks, it seems important to consider that images (i.e., scenes) require longer trials. Compared to faces (from the same model), scenes are more complex visual stimuli with a much wider range of objects and stimulus constellations shown. Scenes require a longer period of time to process and recognize their contents. This could be a reason why there is a longer initial attention allocation to threatening scenes during the first seconds, which has not been found for angry faces. Efficiency of processing facial expressions of basic emotions is illustrated by the fact that the quality of briefly presented emotional faces can be processed even if participants are not aware of their presentation (81, 82). If one is interested in investigating temporal changes in positive attentional bias over a trial, the presentation of four stimuli seems more suitable than that of two stimuli (a positive stimulus combined with a neutral one) and (naturalistic) images seem more suitable than faces. It can be assumed that in multiple-stimulus presentations positive faces attract the attention of viewers much faster than positive images (scenes). The relatively late prioritization of positive images should offer better opportunities to capture the temporal dynamics of the attention allocation processes and interindividual differences in the time course. To assess positive attentional biases relevant for mood protection, it may be important to administer arrays in which positive stimuli are presented along with negative stimuli (and not only with neutral ones). The presence of negative facial expressions in the perceptual field may trigger or reinforce the need for mood regulation. Possibly, the orientation toward positive information in the face of negative information represents an important mood-protective attentional mechanism. It remains to be examined whether the magnitude of a positive bias measured by simultaneously presenting positive and negative stimuli has a greater predictive value with regard to positive mood and depressive symptoms than a positive bias measured by presenting positive stimuli combined with neutral stimuli.

The free-viewing task appears to be particularly suited to capturing interindividual differences in mood-congruent effects on attention. Memory or evaluation tasks that require participants to purposefully attend to each image, regardless of image type, could lead to a more uniform distribution of attention across stimuli that might attenuate individual differences in attention allocation (83). Recently, Sun et al. (84), used simultaneous eye-tracking and fMRI measurements to assess emotional attentional biases in depressed and non-depressed individuals. Gaze behavior during emotion recognition was compared with gaze behavior during free viewing based on the same stimuli. A mood congruent pattern was found in depressed patients only in the free-viewing condition. Emotion recognition was linked to greater activation of the primary visual cortex, whereas free viewing was more strongly associated with activity in the dorsolateral prefrontal cortex. Emotion identification may lead to a more feature-based visual processing while free viewing involves more spontaneous, self-generated attentional responses depending on individual state and trait characteristics.

Individual factors that can influence the magnitude of positive attentional biases and should be considered in future research are trait happiness, alexithymia, experiences of abuse during childhood, habitual use of reappraisal (as emotion regulation strategy), and age. With increasing age, positive stimuli seem to capture attention more quickly and to hold it for longer. It should be clarified whether early onset or the extent of increase in attention to positive information during free viewing are better predictors of positive mood or absence of depressive symptoms than positive bias scores averaged over the task. Future research should investigate whether positive attentional biases are related to adaptive strategies of emotion regulation such as acceptance and problem-solving (85), and other cognitive positivity biases (e.g., in memory processes (5)) and how they interact to protect or improve mood. In this research context, mediation analyses could be a valuable tool to investigate whether preference for positive stimuli forms an attentional pathway through which adaptive emotion regulation strategies (such as cognitive reappraisal or acceptance) promote positive mood and buffer the development of negative affect (86).

Clinical studies investigating positive attentional biases have so far focused on depressive and (social) anxiety disorders. Eye-tracking research into positive attentional biases in other mental health conditions, such as schizophrenia and post-traumatic stress disorder, is less developed. Therefore, future research efforts using the free-viewing task can be directed, for example, towards clarifying whether anhedonia symptoms in schizophrenia or impaired emotion regulation in PTSD are associated with reduced spontaneous attention allocation to positive stimuli.

A novel method that was developed as an alternative to eye tracking is MouseView.js, which uses the computer cursor as a proxy for gaze instead of a webcam (87). This program mimics the visual system’s foveal clarity and peripheral blur by allowing participants to move an aperture on an obscured field with the mouse. There is evidence that MouseView.js approximates results from eye tracking when participants engage in visual exploration of stimuli during free-viewing tasks (87). MouseView.js is a reliable measure of attentional preferences and can replace eye tracking, especially in web-based psychological experiments. Recent studies on the effects of anxiety disorders and sexual orientation on perception demonstrated the utility of MouseView.js for capturing attentional biases (88, 89).

So far, free-viewing eye-tracking studies on attention biases using multiple-stimulus arrays have almost exclusively used static stimuli, i.e., images of faces or images of scenes or objects. Static images cannot adequately depict the dynamic nature of real-life situations and interactions. The use of dynamic stimuli (videos) could strengthen research on attention biases with regard to ecological validity of findings. In a recent eye-tracking study on attention bias in young adults (90), positive, negative, and neutral video clips depicting social situations were shown in side-by-side pairs. Individuals with greater anxiety were found to spend more time gazing at negative compared to paired neutral videos. However, even if videos generally seem promising for attention research, it must be ensured that the amount and speed of motion in the video clips are comparable across different experimental conditions. This represents an important methodological challenge for future research on attentional biases in which videos instead of images are administered.

Future research into positive attentional biases should not only rely on faces or pictures as stimuli but could also include news websites that consist of positive and negative articles. Rudich-Strassler et al. (91) emphasize the importance of ecological validity in attentional research which can be increased through the use of internet websites that resemble real-world online environments.

## Data Availability

The original contributions presented in the study are included in the article/supplementary material. Further inquiries can be directed to the corresponding author.
